# A Bogus Directory

**Published:** 1884-09

**Authors:** 


### A Bogus Directory.

A so-called "Directory of Dentists in the United States," purporting to be published by a party in New York, proves upon a casual examination to be the greatest humbug and most consummate fraud that has of late been perpetrated upon the dental profession and manfacturers of dental goods. It is defective in every aspect (except the advertisements it contains), and full of errors from beginning to end. I learn that the publisher of this work promised great things, but that his promises amount to naught. What I have to say regarding this so-called dental directory, I utter from personal knowledge and know to be true. In the city of its publication there are dentists whose names do not appear, and scores of others whose addresses are incorrect. One town in New York, which has generally four or five dentists, is omitted entirely, whilst I find three towns (with one dentist located at each), which have no post-office and some of them are said to have no existence. In Pennsylvania there are five towns having eight dentists (three being put down at one) which have no post-office and are not found in our geography. In Kansas there is one town to which he gives *three* dentists—there has never been less than *six* permanently located there for several years past. In another town in the same State he puts down *seven* dentists, and it never had more than *three*. Some of the States are short *one-third* to *one-fourth* their actual numbers, and in two States and several Territories he only has about one-half the number. In Chicago and other large cities some of the most prominent dentists are left off the list. In Delaware there are *thirty-two* dentists—this *bogus* directory gives twenty-six, and only fifteen of these are correct, some of the others being dead and removed away three or four years ago. In Maryland the list seems about the same, not over one-half of the addresses can be relied on as being correct, many that are on the list have removed from the State two to five years ago, and new ones located. Altogether the directory part is a farce, almost every dental manufacturer or depot has a better and more perfect list, for I have had the privilege of examining and comparing some of them. It also contains addresses of a large number in over twenty States who have been *dead* for from one to five years, for dentists die occasionally, same as other people. One territory that has several dentists is not given at all. In his "table of dentists," he gives one State as having *seventy-five*, but in his directory only gives addresses of *eleven*, and in one Territory he gives the number as *thirty-four*, but his names only show *four*.

I could give many more comparisons, but these will suffice to show the false character of this work. The pretended list of "dental depots in the United States," is another *farce*, as seven or eight of the largest and best known dealers in the Western States and on the Pacific coast, also those in Maryland and some other Southern States, are left out, whilst he has some who are not known as dealers, and one firm who has been out of business for three years—in fact, he does not give sixty dental depots, when there are known to be nearly or quite one hundred who are legitimately in the trade in this country.

Probably the most interesting part of his work is *twenty-three full* and *five partial pages* of *statistical matter* and *editorial notes* taken from the first edition of "CAULK'S DENTAL ANNUAL," without *giving any credit* whatever, except in one contributed article. Now had this plagiarist, this purloiner of the work of another man's brains, taken the matter from the second edition (published in May) of "CAULK'S DENTAL ANNUAL," instead of the first, he would then have obtained some later and more satisfactory statistics. D.

